# Energy Consumption Research of Mobile Data Collection Protocol for Underwater Nodes Using an USV

**DOI:** 10.3390/s18041211

**Published:** 2018-04-16

**Authors:** Zhichao Lv, Jie Zhang, Jiucai Jin, Qi Li, Baoru Gao

**Affiliations:** 1 Acoustic Science and Technology Laboratory, Harbin Engineering University, Harbin 150001, China; lvzhichao@hrbeu.edu.cn (Z.L.), leechi319@163.com (Q.L.); 2 First Institute of Oceanography, State Oceanic Administration, Qingdao 266100, China; zhangjie@fio.org.cn; 3 College of Engineering, Ocean University of China, Qingdao 266100, China; gaobaoru2010@163.com

**Keywords:** Unmanned Surface Vehicle, data collection, protocol design, energy consumption index

## Abstract

The Unmanned Surface Vehicle (USV) integrated with an acoustic modem is a novel mobile vehicle for data collection, which has an advantage in terms of mobility, efficiency, and collection cost. In the scenario of data collection, the USV is controlled autonomously along the planning trajectory and the data of underwater nodes are dynamically collected. In order to improve the efficiency of data collection and extend the life of the underwater nodes, a mobile data collection protocol for underwater nodes using the USV was proposed. In the protocol, the stop-and-wait ARQ transmission mechanism is adopted, where the duty cycle is designed considering the ratio between the sleep mode and the detection mode, and the transmission ratio is defined by the duty cycle, wake-up signal cycles, and USV’s speed. According to protocol, the evaluation index for energy consumption is constructed based on the duty cycle and the transmission ratio. The energy consumption of the protocol is simulated and analyzed using the mobile communication experiment data of USV, taking into consideration USV’s speed, data sequence length, and duty cycle. Optimized protocol parameters are identified, which in turn denotes the proposed protocol’s feasibility and effectiveness.

## 1. Introduction

Underwater Acoustic Sensor Networks (UWSNs) play a vital role in the process of exploring and developing marine resources, and have gradually become a popular research topic in the field of ocean studies. The underwater observation sensor nodes have long data recycling cycles and expensive problems, such as the seabed base and underwater buoy [1]. With the development of UWSNs communication technology, a sensor monitoring node with acoustic communication has the ability of long-distance and underwater data wireless transmission. However, the methods of data collection of underwater nodes are mainly based on the survey ship and buoy relay. The survey ship has a high operation cost and low data collection efficiency. The system of buoy relay is complex and inflexible.

The unmanned platforms and related technologies are increasingly matured and perfected, which can provide support for the underwater acoustic data collection [2,3]. In particular, the USV integrated with an underwater acoustic modem has some advantages such as easy integration, fast laying, and a low cost, and has become an alternative novel node for underwater networks. Researchers at the University of Porto developed the unmanned ship ZARCO to carry out communication and positioning research among Autonomous Underwater Vehicles (AUV) [4]. Engineers from Massachusetts Institute of Technology used the underwater communication among several USVs by underwater communication acoustic modems, which developed a communication and positioning system of USVs [5]. The USV which was developed at the Japan Naval Research Center in 2015 completed a joint images transmission experiment with an AUV [6]. The developers of the Lisbon Institute of Advanced Technology (IST) and Ocean Robot (DSOR) in Portugal developed DELFIMX, which was originally used as an acoustic relay between an AUV and auxiliary boat [7,8]. The USVs in the above papers were applied in point-to-point underwater acoustic communication, which was influenced by a time-varying channel and Doppler effect, and few applications have been conducted in UWSNs.

In the field of underwater acoustic network research, we have already had some large international supporting programs, such as the Seaweb plan, but mature underwater acoustic networks based on a mobile platform have been very few in number. Vasilescu et al. developed an underwater data collection system for mobile and stationary nodes based on optical and underwater acoustic communications, where the accesses method was the relay node communicating with an AUV [9]. Cheng and Li [10] considered data importance and energy consumption in underwater wireless sensor networks, where an AUV and muti-hop transmission were used. Free competition and its extension method were applied to the underwater sensor network, where optimization was achieved mainly by compensating for the underwater acoustic delay in ad hoc networks [11,12]. The research of access communication protocol between nodes and carriers was seen in the research of data donkey protocol. Boloni and Turgut developed a deterministic data access protocol for the random move mode of the data donkey [13]. Anastasi [14] presented an adaptive data access protocol that took the packet loss rate into account. Although there are already many applications in the mobile platform MAC protocol, the impacts of the underwater acoustic channel and energy optimization of protocol have rarely been considered. Furthermore, the design for data collection protocol should consider the impact of the current underwater environment and the difficulties faced by the application scenario.

In this paper, we propose the aim to extend the life of UWSNs and optimize the energy consumption of the nodes and a mobile data collection protocol for underwater nodes using USV. The energy parameters of the nodes are optimized to support the optimization of the underwater sensor network structure and the unmanned data collection. The remainder of the paper is structured as follows. In Section 2, we describe the application scenario of a USV collecting underwater sparse network data. In Section 3, we conduct the node energy consumption analysis. Section 4 presents the design of data collection protocol and Section 5 concludes our work and further discusses some future work.

## 2. The Application Scenario of USV Collecting Underwater Sparse Network Data

In the field of marine monitoring, a sparse monitoring network needs to be set up, in order to save costs and expand the monitoring area. The sensor node design mainly considers the node life, the data transmission, and other factors, which are related to the energy. In sparse networks, the distance among nodes is relatively large, and communication among nodes is relatively difficult due to energy constraints.

**Figure 1:**
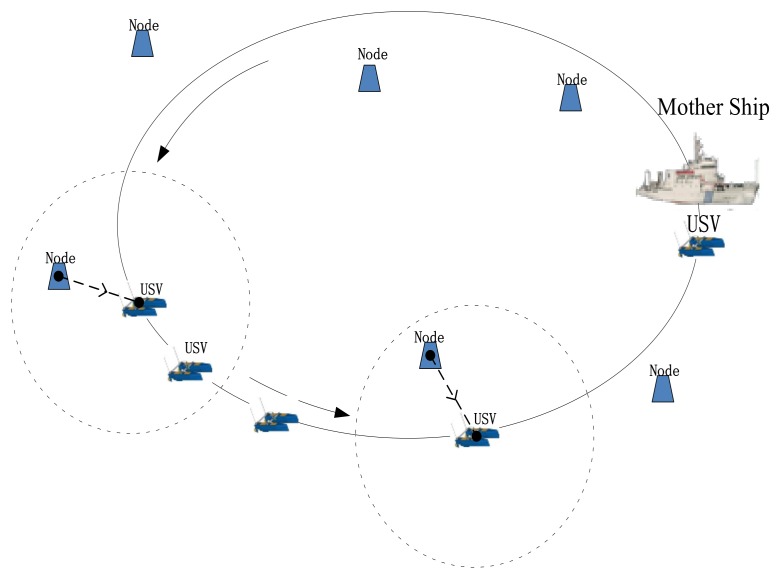
Sparse network data collection schematic.

In order to save energy and complete the long-term underwater monitoring tasks, we implement the method of USV mobile collecting nodes data instead of the method of inter-nodes underwater acoustic communication, as shown in Figure 1.

In this scenario, the USV starts at the mother ship, sails along the pre-planned path of self-navigation, communicates with the adjacent nodes, and collects the monitoring data of the node which will be stored locally or sent to the mother ship immediately through the wireless radio. This application scenario mainly considers the energy consumption of the nodes in the underwater acoustic network without taking into account the energy consumption of the USV nodes. In the ideal case, based on the marine environment and the node energy consumption, the USV will autonomously choose modulation speed and communication protocol parameters.

## 3. Analysis of Node Energy Consumption

In the sparse acoustic network, the USV collects underwater data using point-to-point communication. Regardless of the energy consumption of the USV, node communication consumption is the main energy consumption of sparse networks. We analyze the node energy consumption model, as follows [15]:

(1) $$E_{\mathrm{tran}}\left(i\right)=\left\{ \begin{array}{l} L_{d}\varepsilon_{elec}+L_{d}a{\left(f\right)}^{d}d{\left(i\right)}^{2},d\left(i\right)<d_{0}\\ L_{d}\varepsilon_{elec}+L_{d}a{\left(f\right)}^{d}d{\left(i\right)}^{4},d\left(i\right)\geq d_{0} \end{array}\right.$$

(2) $$E_{\mathrm{reve}}\left(i\right)=L_{d}\varepsilon_{elec}$$

Equations (1) and (2) are used to calculate the transmission energy and the reception energy consumption of the communication between the node *i* and USV, respectively. $L_{d}$ is the single packet length. $\varepsilon_{elec}$ is the transmission and reception power consumption of circuit running. $\;a{\left(f\right)}^{d}$ is the amplification factor. $d_{i}$ is the distance between the node and USV, and $d_{0}$ is the node communication distance threshold. a(f) is the absorption coefficient, and f is the acoustic signal frequency. $E_{tran}$ is the energy consumption of the transmission, and $E_{reve}$ is the energy consumption of reception. In the high frequency band of communication, the loss can be expressed as [16]:

(3) $$\log a\left(f\right)=0.011\frac{f^{2}}{1+f^{2}}+4.4\frac{f^{2}}{4100+f^{2}}+2.75\times 10^{-5}f^{2}+0.0003$$

It can be seen from the node energy consumption model that both the transmission consumption and the reception consumption are related to the packet length and power, but the transmission energy is obviously higher than the reception energy. The further the transmission distance, the higher the consumption energy. It is of importance to reduce the consumption of node communication and avoid the packet retransmission caused by conflicts or mistakes of the packet.

### 3.1. The Design of Data Collection Protocol and Energy Consumption Index

**Figure 2:**
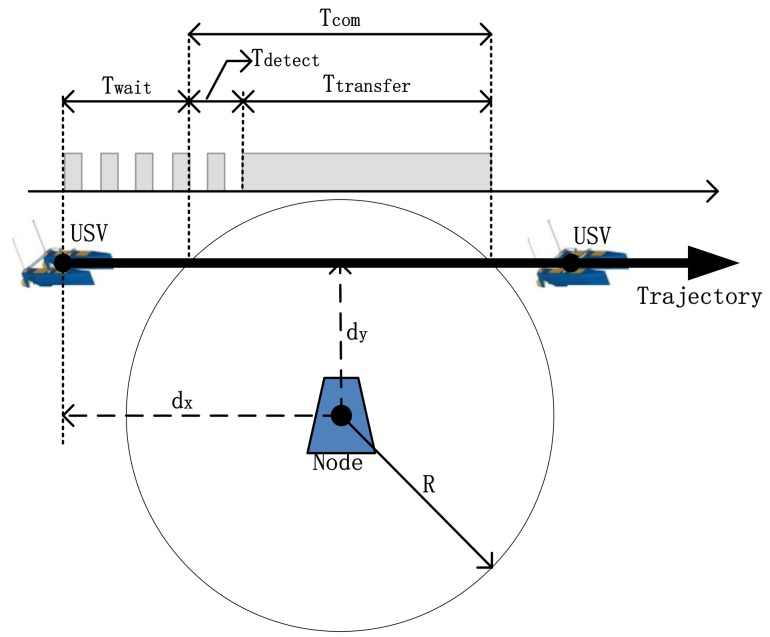
Point to point communication applications diagram.

In the above scenario, acoustic communication is used to collect node data within an effective communication distance. The communication range is a circle with the center at the node position and the radius *R* for the communication radius. The point-to-point data communication applications are shown in Figure 2.

Figure 2 shows that the USV travels at a constant speed through the communication range of node and the navigation trajectory can be approximated by the vertical distance of $d_{y}$ centered on the node. The communication process can be roughly divided into a detection phase and transmission phase. At the time of data collection, the USV periodically sends a wake-up signal, and the nodes’ mode is between the sleep mode and the detection mode. When the node detects the USV in the communication range, it converts to the transmission phase, and the transmission mode is fully open-designed.

**Figure 3:**
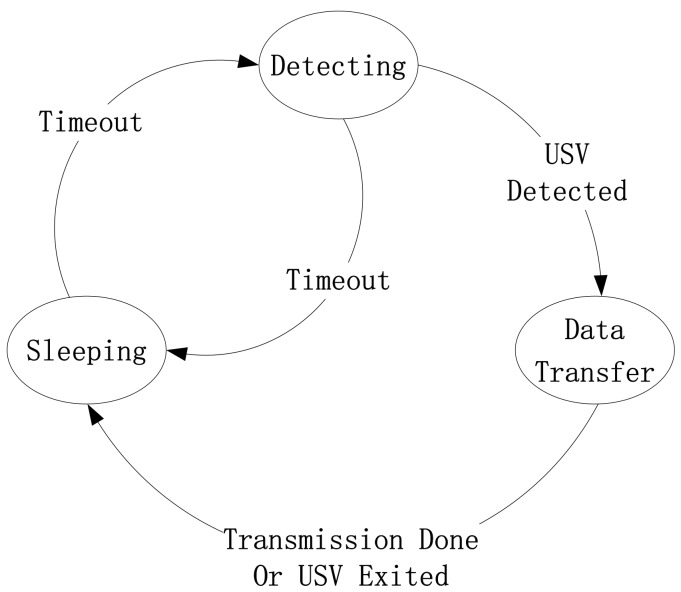
Diagram of node state transition.

As shown in Figure 3, the node status can be divided into three categories: sleep mode, detection mode, and transmission mode. The initial state is the detection mode, and it periodically switches between the sleep mode and the detection mode. Once the USV is detected in the scope of its communication, the transmission mode starts. Besides, when the data transfer is completed or the USV exceeds the node communication range, the data transmission ends and the state transition is turned to the sleep mode. When the USV starts to collect the sparse network monitoring data from the mother ship in the next period, the detection phase starts again.

Based on the three modes, the ratio between the sleep mode and the detection mode, and the overall transmission efficiency for the communication are considered. So, the designed duty cycle and defined transmission ratio are used to construct the evaluation index for energy consumption.

### 3.2. Design of the Duty Cycle

During the detection phase, the USV periodically broadcasts the awaken signal, whose period is T, and its waking packet duration is $T_{w}$. We define the duty cycle δ, which can be expressed as follows:

(4) $$\delta =\frac{T_{on}}{T_{on}+T_{off}}=\frac{T_{w}+\;T_{delay}+T}{T_{on}+T_{off}}$$

where $T_{delay}$ is the delay of the communication distance, and $T_{on}$ is the duration of the detection mode. It consists of awakening signal duration, the wake-up signal cycle, and time delay, which are mainly designed to ensure that the node completely captures wake-up signals sent by the USV when the USV moves into the node communication range. For the duration of the sleep mode, the values are set to $T_{on}$ by δ jointly.

When the wake-up signal sent by the USV is captured by a node, the transmission phase will start, that is, its conversion immediately turns to the transfer mode. The fully open duty cycle is designed to minimize the transmission time of the data communication and reduce the possibility of break, so as to increase the transfer efficiency.

**Figure 4:**
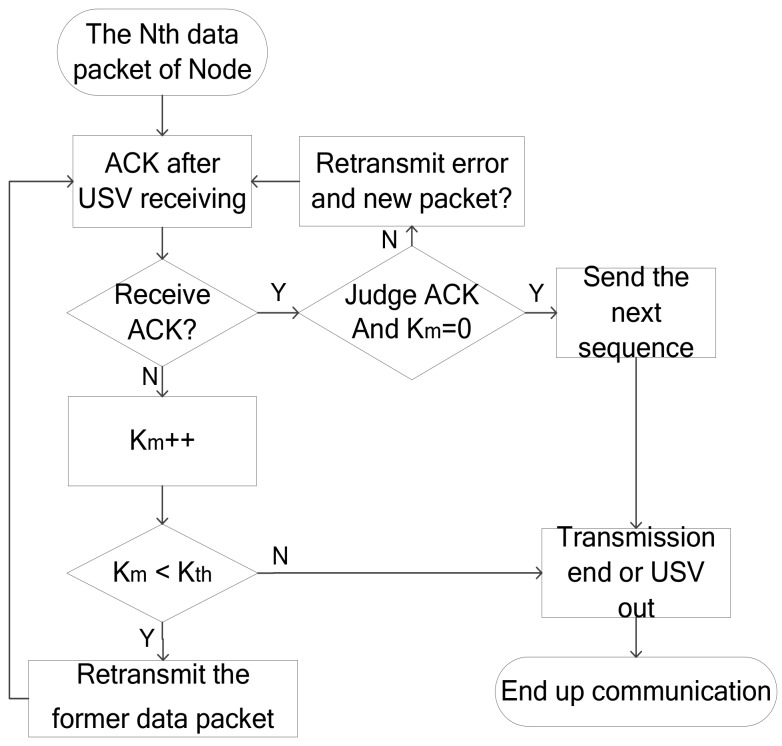
Flow diagram of data collection protocol.

As shown in Figure 4, the USV converts to the receiving mode. Instead of sending the wake-up signal, the USV responds to the ACK signal after receiving the node packet. The point-to-point communication between the node and the USV uses a stop-and-wait ARQ transmission mechanism. The nodes send data sequence including N packets. The duration of each packet $T_{d}$ is fixed, and N is the same for the USV and nodes, which is known as the window size. After the node sends the packet, it will wait for the ACK signal response of the USV, and the ACK signal encapsulates the information whether or not the $K_{th}$ packet is transmitted correctly. The ACK signal duration is $T_{ack}$. If the node does not receive an ACK signal from the USV in $2T_{delay}+T_{ack}$, the data sequence will be resent and $K_{m}$ will be incremented by one. If it receives an ACK signal from the USV again, $K_{m\;}$ resets to 0. When $K_{m\;}$ reaches the threshold value$\;K_{th}$, the USV is already out of the node communication range or the data transfer is completed, hence ending the transmission phase.

### 3.3. Transmission Ratio

In the protocol, the transmission ratio is an important indicator of energy consumption. The larger the transmission ratio, the longer the effective communication time between the USV and the node. The transmission ratio is determined by the duty cycle, wake-up signal cycles, and USV’s speed. The transmission ratio can be defined as follows:

(5) $$\varsigma =\frac{T_{transfer}}{T_{com}}\left(1-\theta \right)$$

where $T_{com}\;$ is the time when the USV is within the nodes communication range, and $T_{transfer}$ is the timespan from when the node and USV are communicating point-to-point to when data transmission is finished or USV sails out of the communication range. θ is the packet error rate, which will be analyzed in the later communication experiment. The transmission ratio will be analyzed in the later simulation experiments.

### 3.4. Design of the Energy Consumption Index

In order to save the sparse network energy, the duty cycle needs to be reasonably designed. If the duty cycle is too small, the data transmission may be missed, which cannot satisfy the needs of the data collection. If the duty cycle is too large, it will waste more energy and will not meet the requirements of energy efficiency. In order to evaluate the energy consumption of the node, we consider the ratio between the sleep mode and the detection mode, and the overall transmission efficiency for communication. The energy consumption index is defined as follows:

(6) $$\;\;E_{byte}=\frac{P_{sleep}\cdot T_{sleep}+\;P_{re}\cdot T_{re}+P_{tr}\cdot T_{tr}}{n_{bytes}}$$

where$\;P_{\mathrm{sleep}\;}$, $P_{\mathrm{re}}$, and$\;P_{tr}$ represent the power consumption of node in the sleep mode, the detection mode, and the transmission mode, respectively; and the durations for the three modes are $T_{sleep}\;$, $T_{re}$, and $T_{tr}$, respectively, by Equations (7)–(9). In Figure 2, $T_{delay}^{*}$ is the transmission time delay between the node and the USV, and it slightly varies with the change of the communication distance. Since the USV is sailing straightly and is close to the boundary of the communication area of the nodes, $T_{delay}^{*}$ can be approximately seen as $T_{delay}$*.* $T_{wait}$ is the timespan from when it turns from the node state to the detection state to when it sails to the communication boundary. $T_{detect}$ is the timespan from when USV sails into the node communication range to when it is actually detected by the node.

(7) $$\;\;\;T_{sleep}=\left(T_{wait}+T_{detect}\right)\cdot \left(1-\delta \right)$$

(8) $$T_{tr}=\left(\frac{N\cdot T_{d}}{N\cdot T_{d}+2\cdot T_{delay}^{*}+T_{ack}}\right)$$

(9) $$T_{\mathrm{re}}=\left(T_{\mathrm{wait}}+T_{\mathrm{detect}}\right)\cdot \delta +\left(\frac{2\cdot T_{\mathrm{delay}}^{*}+T_{\mathrm{ack}}}{N\cdot T_{d}+2\cdot T_{\mathrm{delay}}^{*}+T_{\mathrm{ack}}}\right)\cdot T_{\mathrm{transfer}}$$

By putting Equations (7)–(9) into Equation(6), the energy consumption index has been rewritten as a function of two important factors ς and δ in Equation(10). This energy consumption index will be evaluated and discussed in Section 4.

(10) $$E_{byte}\;=\;A\left(P_{re}-P_{sleep}\;\right)\delta +\left(BP_{tr}+\;CP_{re}-BP_{re}\right)\varsigma +AP_{sleep}\;$$

where A,  B, and C are defined as follows:

(11) $$A\;=\frac{T_{wait}+T_{detect}}{n_{bytes}}$$

(12) $$B\;=\;\left(\frac{N\cdot T_{d}}{N\cdot T_{d}+2\cdot T_{delay}^{*}+T_{ack}}\right)\frac{T_{com}}{n_{bytes}}$$

(13) $$C=\frac{T_{com}}{n_{bytes}}$$

In the following analysis of data collection energy consumption, the speed of the USV, the data sequence length, and the duty cycle can be simulated, but the packet error rate must be acquired from the experiment. In the following experiment, the packet error rate was calculated by the received data and used in the simulation experiments.

## 4. Analysis of Data Collection Energy Consumption of USV

Aiming at evaluating the underwater acoustic communication ability between the USV and underwater node, the underwater acoustic communication experiment for the USV is carried out in a lake, where the parameters such as distance underwater acoustic communication and the communication packet error rate are considered. Based on the data from the experiment, the simulation experiment is carried out, and influences caused by USV speed, data sequence length and duty cycle on protocol performance, and energy consumption are analyzed.

### 4.1. Underwater Acoustic Communication Experiment for USV

**Figure 5:**
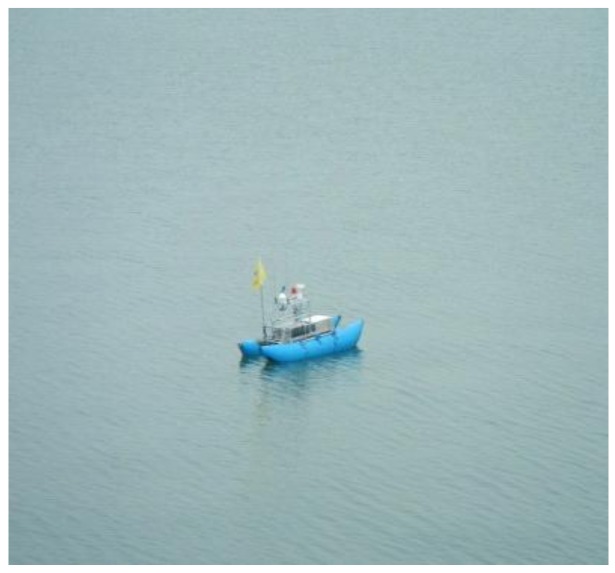
The unmanned surface vehicle “USBV”.

The lake experiment was carried out for the underwater acoustic communication at the JiHongTan reservoir in Qingdao on 15 July 2015 (Figure 5). The circumference of the reservoir is about 14 km, and the mean depth is about 10 m. A pair of EvoLogics underwater acoustic modems are used in the experiment, where one modem is set underwater 2 m at the shore, and the other is carried on the USV platform as a mobile node at a depth of 3 m. Additionally, an icListen hydrophone is installed at a depth of 1 m on the USV, which monitors communication signals with a sampling frequency of 256 kHz.

**Figure 6:**
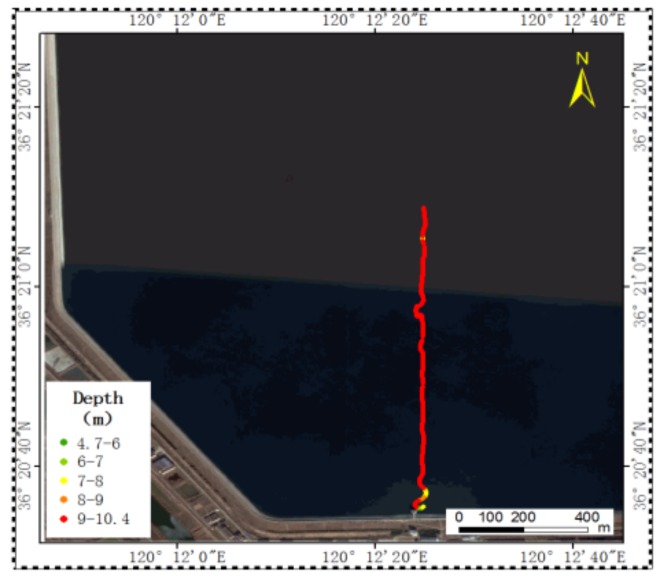
Mobile communication’s trajectory of USV.

In order to verify the mobile underwater acoustic communication ability of the USV, the desired trajectory is set as a straight line in the north direction, with the starting point 36°20′35″ N, 120°12′25″ E and the end point 36°21′12″ N, 120°12′25″ E. Using the autonomous control of the USV, the expected line is followed automatically for 1 km, which can seen in Figure 6.

**Figure 7:**
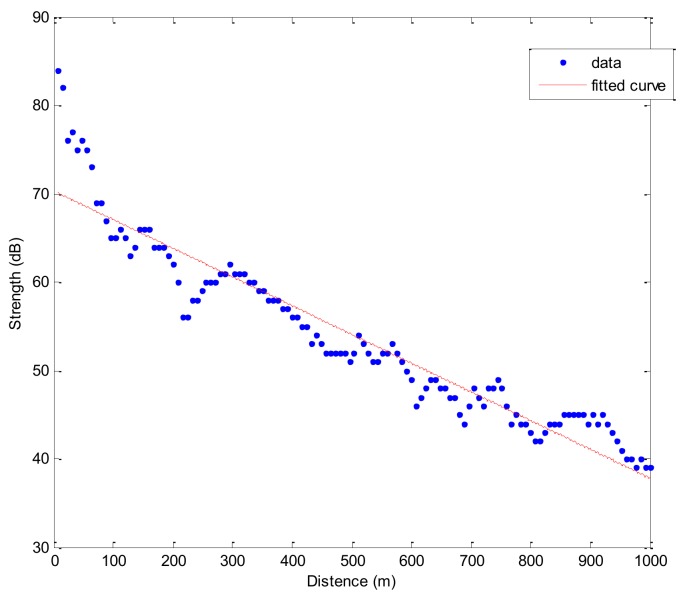
Signal strength varying with distance.

**Figure 8:**
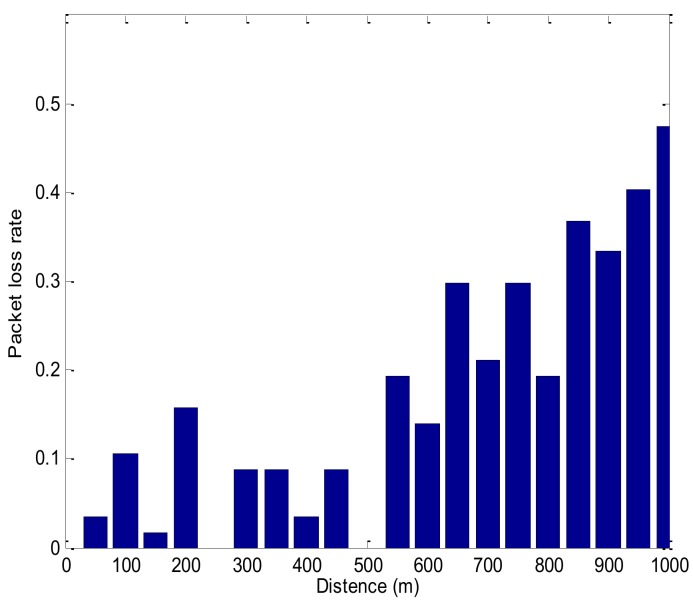
Packet error rate varying with distance.

As shown in Figure 7, the blue star indicates the signal strength and the red line is the fitted curve. The communication abilities between communications nodes become weaker when the communication distance increases. In the experiment, the maximum communication distance is 1.9 km. In order to guarantee the success rate of data transmission, the communication radius is set as 1 km in the following protocol simulation. In sparse underwater monitoring networks, USVs communicate with the neighboring nodes point-to-point with no packet collisions. However, as a result of the unstable underwater acoustic communication channel, there may be packet errors, which can lead to packets retransmission between nodes, and affect the lifespan of the sparse network. In Figure 8, the packet error rate is averaged by 53 sets of data, and it increases with the communication distance. So, the greater the distance between the node and the USV, the greater the number of retransmissions and the energy consumed. When the distance is 200 m, the packet loss rate suddenly increases, which corresponds to the sudden decrease of the signal strength in Figure 7. The change of packet error rate with distance is used to support the simulation in the detection phase and transmission phase.

### 4.2. The Simulation of the Detection Phase

**Figure 9:**
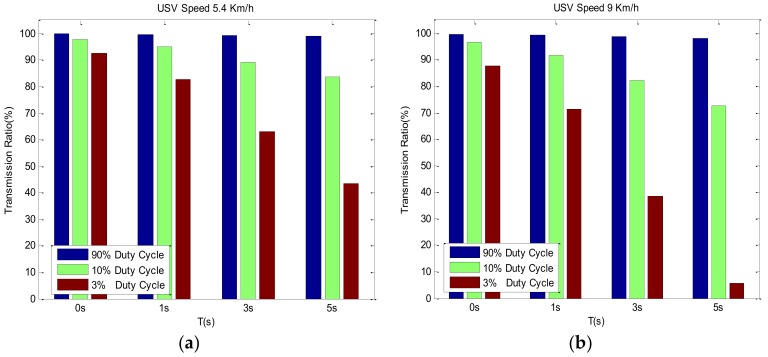
Simulations of transmission ratio.

In Figure 2, we set the following parameters, R=1 km,  dy=32R. According to the energy consumption of the acoustic Modem Evologics S2CR series, the node powers of sleep mode, detection mode, and transmission mode are 0.2 W, 1.3 W, and 18 W, respectively. Both the wake-up signal and ACK response signal length are 72 bits, and the packet length sent by a single node is 1024 bits. The data rate of packet transmission is 976 bps. The packet error rate results from an underwater acoustic communication experiment. During the detection phase, the transmission ratios of different USV wake-up signal cycles and different duty cycles are simulated, as shown in Figure 9.

In Figure 9a,b, different USVs’ wake-up signal cycles and transmission ratios in different protocols are shown with the speed of the USV is 5.4 km/h and 9 km/h, respectively. The larger the ratio of the agreement, the greater the transmission ratio. Additionally, the effective communication time between the USV and nodes is increased, which means the node can detect the USV in its communication range in time. During the detection phase, the main factors that influence ς are T and δ. Comparing Figure 9a,b, it can be seen that the smaller the USV’s speed is, the greater ς is, which means the USV is slower, and the node can detect USV more easily in time under the same conditions. In Figure 9a, even when T=5 s and the duty cycle is 3 % , it can still reach 43%, indicating that when the USV’s speed is 5.4 km/h, the node can effectively send the monitoring data with a lower energy consumption.

In Figure 9b, when the USV runs faster, the available communication time of the node becomes shorter. When T is invariant, the smaller the δ is, the longer the nodes’ sleep mode is, the longer the waiting time for the USV, the shorter the time left for communication between the USV and nodes, and therefore, ς is smaller. Obviously, when T continues to grow and δ continues to decrease, a special case may occur in which the nodes are unable to detect the USV.

### 4.3. The Simulation of the Transmission Phase

**Figure 10:**
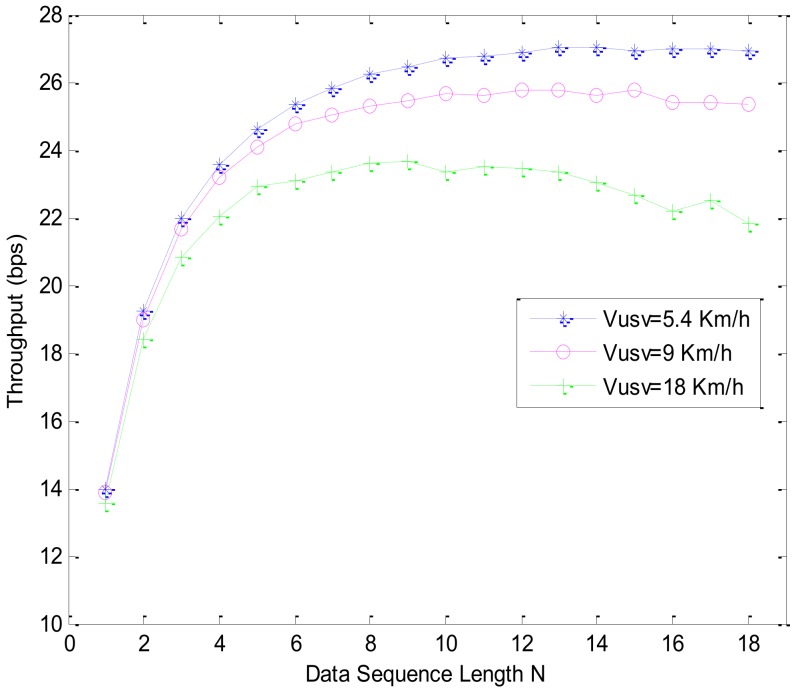
Protocol throughput simulation for data sequence length *N* and velocity of USV.

**Figure 11:**
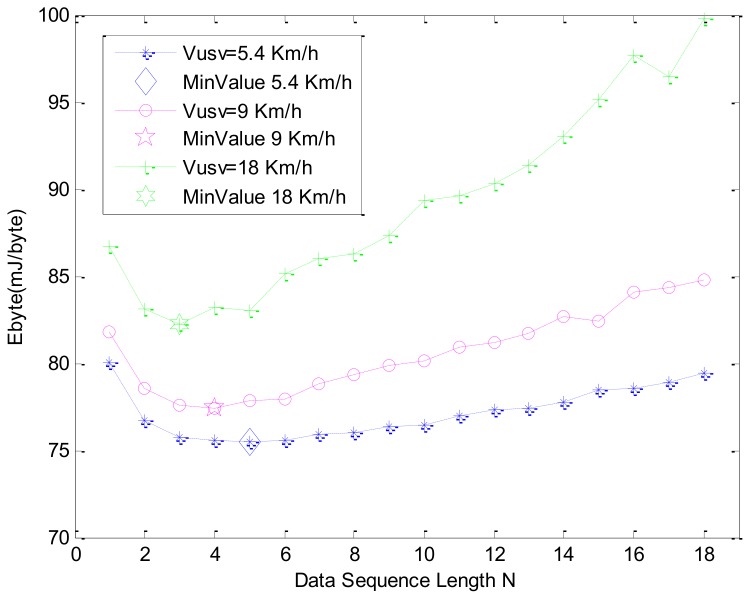
Energy consumption simulation for data sequence length *N* and velocity of USV.

During the transmission simulation, the protocol throughput and node energy consumption of different data sequence lengths N and different USV speeds are studied. On the basis of the detection simulation, we select the *T* as 3 *s* and duty cycle as 10%. The protocol throughput simulation and energy consumption simulation are shown in Figure 10 and Figure 11, respectively.

Figure 10 shows the protocol throughput in different data sequence lengths N and the USV’s velocity. It can be seen that protocol throughput has an increasing trend along with the increase of the data sequence length N. The bigger the USV’s speed, the smaller the maximum throughput. When the N is small, the ACK signal and the round-trip transmission delays of the USV response node hold the greatest proportion, and the throughput will be increased as the N increases. If N is large, more information needs to be retransmitted and a longer transmission time is required. At this point, the USV may have moved out of the node communication range and failed to receive. Based on the protocol throughput simulation, the energy consumption simulation is executed, which is shown in Figure 11.

**Figure 12:**
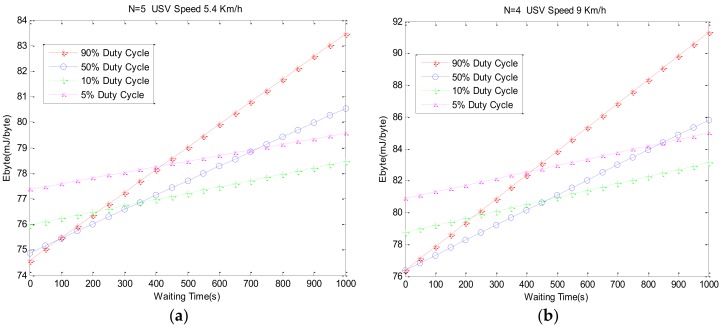
Energy consumption simulation under different waiting times.

Figure 11 shows the unit byte energy consumption at different data sequence lengths *N* and the USV’s velocity. When N increases, $E_{byte}$ decreases firstly and then it increases. When N increases, $E_{byte}$ decreases first and then it increases. The greater the USV velocity is, the greater the$\;E_{byte}$ is. The greater the$\;E_{byte}$ is, the smaller the minimum value of $E_{byte}$ is. This is because the protocol throughput is smaller in the smaller N, and because of the high energy consumption in the detection phase, $E_{byte}$ is larger. When the point-to-point communication is stable, protocol throughput increases, energy consumption is faster, and $E_{byte}$ begins to decrease. The greater the USV’s speed, the smaller the data sequence length. When the USV is faster, the ACK signals are more likely to lose or make mistakes and the $E_{byte}$ increases faster. Under the set of parameters *T* as 3 s and duty cycle as 10%, in order to get the lowest energy consumption, we can choose different data sequence lengths *N* and USV speeds according to the results in Figure 11, which will cope with different application requirements. In the case of actual data collection, the initial position of the USV is difficult to determine, so the${\;T}_{\mathrm{wait}}$ must be considered. Based on the above experiments, when the USV’s speed is 5.4 km/h, the N corresponding to the minimum $E_{\mathrm{byte}}$ value is selected, which is N=5. When the USV’s speed is 9 km/h, N=4*.* The simulation results of energy consumption with different $T_{wait}$ and δ values are shown in Figure 12.

Figure 12a indicates the change diagram of $E_{byte}$ under different $T_{wait}$ and δ value when the USV’s speed is 5.4 km/h. $E_{\mathrm{byte}}$ increases obviously with the increase of $T_{wait}$. But when the $T_{wait}$ is small, the smaller δ is, the larger $E_{byte}$ is. This is due to the smaller $T_{wait}$. When the USV is about to reach the node communications range, in order to get timely detection of the USV in its scope, choosing the larger δ is better, and selecting the smaller δ may miss the connection. Compared with Figure 12a, $E_{byte}$ is roughly the same in terms the change under the different $T_{wait}$ and δ values in Figure 12b, but under the same $T_{wait\;}$ and δ values, the faster the USV sails, the larger the $E_{byte}$, and the energy consumption of each byte is bigger.

In the simulations, a different combination of parameters for minimizing energy consumption can be chosen according to the actual needs. For example, the velocity of the USV is usually low in the poor sea conditions, and a higher data sequence length and lower duty cycle should be considered.

## 5. Conclusions

This paper describes the necessity and importance of the energy efficiency design agreement of underwater acoustic sensor networks’ data collection using USV. Aiming at the situation of an underwater acoustic sparse network, a method for the mobile collection of underwater node data based on USVs is proposed. The duty cycle is designed considering the ratio between the sleep mode and the detection mode, and transmission ratio is defined by the duty cycle, wake-up signal cycles, and the USV’s speed. According to the protocol, the evaluation index for energy consumption is constructed based on the duty cycle and the transmission ratio. By the lake trial and simulation experiment, the USV’s speed, data sequence length and duty cycle, transmission ratio, and other parameters are analyzed and optimized for energy consumption. In the actual data collection process, according to the actual needs, we can choose a different combination of parameters to ensure a minimum energy consumption. In future work, we will continue to research the environment adaptive protocol and the traveling path for the USV.

## References

[1] Qi E., Zhang Y. (2011). Sea marine environment automatic monitoring system of research. J. Mar. Technol..

[2] Yuh J., Marani G., Blidberg D.R. (2011). Applications of marine robotic vehicles. Intell. Serv. Robot..

[3] Basagni S., Bölöni L., Gjanci P., Petrioli C., Phillips C.A., Turgut D. Maximizing the value of sensed information in underwater wireless sensor networks via an autonomous underwater vehicle. Proceedings of the INFOCOM.

[4] Melo J., Matos A. Guidance and control of an ASV in AUV tracking operations. Proceedings of the OCEANS.

[5] Benjamin M.R., Curcio J. COLREGS-based navigation of autonomous marine vehicles. Proceedings of the 2004 IEEE/OES Autonomous Underwater Vehicles.

[6] Suzuki N., Kitajima H., Kaba H., Suzuki T., Suto T., Kobayashi A., Ochi F. An experiment of real-time data transmission of sonar images from cruising UUV to distant support vessel via USV: Development of underwater real-time communication system (URCS) by parallel cruisingOceans. Proceedings of the OCEANS.

[7] Alves J., Oliveira P., Oliveira R., Pascoal A., Rufino M., Sebastiao L., Silvestre C. Vehicle and Mission Control of the DELFIM Autonomous Surface Craft. Proceedings of the 2006 14th Mediterranean Conference on Control and Automation.

[8] Vasconcelos J.F., Silvestre C., Oliveira P. (2011). INS/GPS, Aided by Frequency Contents of Vector Observations With Application to Autonomous Surface Crafts. IEEE J. Ocean Eng..

[9] Vasilescu I., Kotay K., Rus D., Dunbabin M., Corke P. Data collection, storage, and retrieval with an underwater sensor network. Proceedings of the 3rd International Conference on Embedded Networked Sensor Systems.

[10] Cheng C.F., Li L.H. (2016). Data Gathering Problem with the Data Importance Consideration in Underwater Wireless Sensor Networks. J. Netw. Comput. Appl..

[11] Karlidere T., Cayirci E. A Mac Protocol for Tactical Underwater Surveillance Networks. Proceedings of the 2006 IEEE Military Communications Conference.

[12] Molins M., Stojanovic M. Slotted FAMA: A MAC protocol for underwater acoustic networks. Proceedings of the OCEANS 2006—Asia Pacific.

[13] Boloni L., Turgut D. (2008). Should I send now or send later? A decision-theoretic approach to transmission scheduling in sensor networks with mobile sinks. Wirel. Commun. Mob. Comput..

[14] Anastasi G., Conti M., Monaldi E., Passarella A. An Adaptive Data-transfer Protocol for Sensor Networks with Data Mules. Proceedings of the 2007 IEEE International Symposium on a World of Wireless, Mobile and Multimedia Networks.

[15] Wang K., Gao H., Xu X., Jiang J., Yue D. (2016). An Energy-Efficient Reliable Data Transmission Scheme for Complex Environmental Monitoring in Underwater Acoustic Sensor Networks. IEEE Sens. J..

[16] Stojanovic M., Preisig J. (2009). Underwater acoustic communication channels: Propagation models and statistical characterization. IEEE Commun. Mag..

